# Enhancement of Antioxidant and Isoflavones Concentration in Gamma Irradiated Soybean

**DOI:** 10.1155/2013/383574

**Published:** 2013-11-05

**Authors:** Boris M. Popović, Dubravka Štajner, Anamarija Mandić, Jasna Čanadanović-Brunet, Slavko Kevrešan

**Affiliations:** ^1^Faculty of Agriculture, University of Novi Sad, Trg Dositeja Obradovića 8, 21000 Novi Sad, Serbia; ^2^Institute for Food Technology, University of Novi Sad, Bulevar Cara Lazara 1, 21000 Novi Sad, Serbia; ^3^Faculty of Technology, University of Novi Sad, Bulevar Cara Lazara 1, 21000 Novi Sad, Serbia

## Abstract

Serbian soybean genotype Ana was gamma irradiated at doses of 1, 2, 4, and 10 kGy in order to evaluate the influence of gamma irradiation on isoflavone (genistein, daidzein, and their glycosides genistin and daidzin) contents and hydroxyl radical scavenging effect (HRSE). The increase in genistin and daidzin contents as well as antioxidant activities was observed especially at doses of 4 and 10 kGy. Results were also compared with our previous results relating to total phenol content (TPC), DPPH radical scavenger capacity (DPPH RSC), and ferric reducing antioxidant power (FRAP). Our results indicated that doses up to 10 kGy improve the antioxidant activities of soybean and also nutritional quality with respect to isoflavone content. All results were analyzed by multivariate techniques (correlation matrix calculation and autoscaling transformation of data). Significant positive correlations were observed between genistin, daidzin, DPPH RSC, and HRSE.

## 1. Introduction

Legumes play an important role in the traditional diets of many regions throughout the world. Soybean and its processed products have been acclaimed as health foods due to their high content of protein and essential amino acids, omega-3 fatty acids, fat-soluble vitamins, polysaccharides, and insoluble fibers [1]. Besides these constituents, soybeans also contain isoflavones that are of wide interest due to their beneficial effects on humans, such as prevention of cancer, cardiovascular diseases, osteoporosis, and menopausal symptoms [2].

Soybean seeds contain many phenolic compounds such as 5-O-caffeoylquinic acid, caffeic acid, ferulic acid, and *p*-coumaric acid. A form of flavonoid in soybean seeds, isoflavones, has been found to have important secondary compounds with many chemical actions. Isoflavones are categorized chemically by their functional groups. There are four subgroups: aglycones, glycosides, malonyl-glucosides, and acetyl-glucosides [3]. Isoflavone phytoestrogens found mainly in soybeans and clover are widely studied phytochemicals. Genistein and daidzein, the major isoflavones found in soy, have received the most attention. Genistein and daidzein, in soybean, are mainly present in the form of their glycosides. Previous research found that malonyl genistin was the most abundant in soybean, followed by malonyl glycitin, genistin, daidzin, daidzein, genistein, and glycitin in decreasing order [4]. Daidzein and genistein are present mainly in the form of their glycosides and malonyl-glycosides in soybean. Lee et al. [5] showed that the soybean isoflavone glycosides possessed at least a similar antioxidant potency to the two aglycones, genistein and daidzein, when the potency was assessed using the anti-DPPH free radical and FRAP assays.

Gamma irradiation has long been known as a method for food preservation [6]. Ionizing irradiation is nonthermal technology that effectively eliminates food-borne pathogens in various foods [7]. Joint expert committee including World Health Organization (WHO), International Atomic Energy Agency (IAIE), and Food and Agriculture Organization (FAO) has approved doses of ionizing radiation up to 10 kGy for treatment of seeds intended for sprout production to inactivate human pathogens [8]. So far, the influence of gamma irradiation (doses up to 5 kGy) on soybean isoflavones and free radical scavenger capacity was investigated also by Variyar et al. [9] and Dixit et al. [10]. In our previous work [11] the effect of doses up to 10 kGy on antioxidant characteristics of Serbian soybean genotype Ana was investigated. The increase of total phenolic and tannin contents and DPPH scavenger activity and the decrease of protein oxidation intensity were established. The aim of our study was to investigate the effect of gamma irradiation (doses up to 10 kGy) on isoflavone contents and hydroxyl radical scavenging effect (HRSE) in Serbian genotype Ana and to compare them with our previous results concerning phenolic content and DPPH radical scavenger activity.

## 2. Material and Methods

Soybean seeds (*Glycine max *(L.) Merr.) of yellow coated genotype Ana were obtained from the Institute of Field and Vegetable Crops in Novi Sad. Soybean seeds were irradiated with the following doses of gamma radiation: 1, 2, 4, and 10 kGy. For that purpose the ^60^Co was used. The dose rate was 228 Gy/min. Seed irradiation was performed in the Laboratory for Radiation Chemistry and Physics “Gama” at the Institute of Nuclear Sciences in Vinča, Belgrade. Irradiated and nonirradiated soybean seeds (100 seeds) were ground in a mill and reduced to a fine powder. Description of the determination of total phenol and tannin contents, DPPH radical scavenging capacity, and FRAP was described in our previous work [11].

### 2.1. Hydroxyl Radical Scavenging Effect (HRSE) Determination

**Figure 1:**
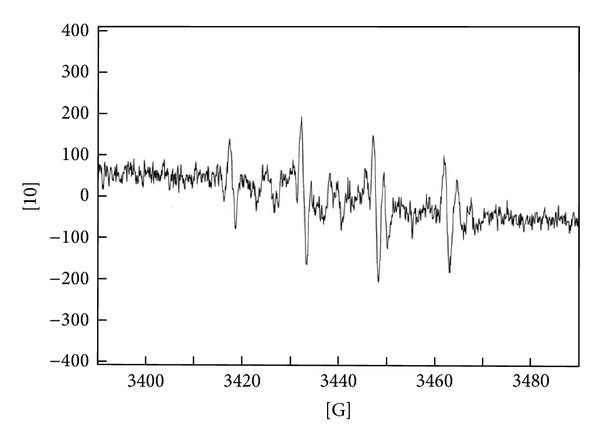
ESR spectra of DMPO-OH spin-adduct model system in the presence of ethyl-acetate extract of soybean irradiated with 10 kGy.

**Figure 2:**
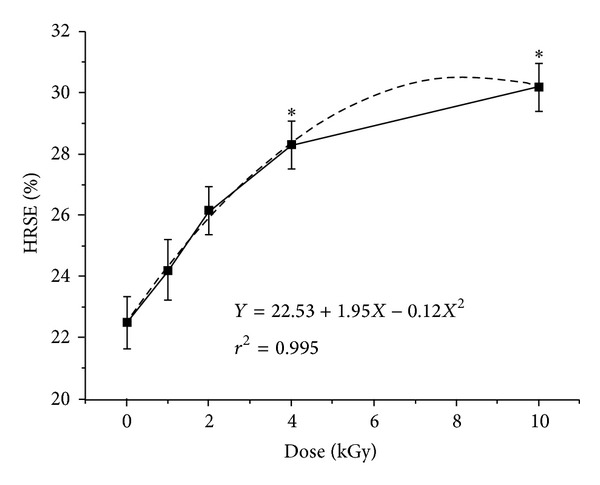
Effect of different doses of gamma irradiation on HRSE of soybean extracts.

The influence of the soybean ethyl-acetate extract on hydroxyl radical (HO^•^) formation was studied by electron spin resonance (ESR) using a spin trapping method [12]. Previously prepared 70% aqueous ethanolic extracts were evaporated to dryness and then dry residues were redissolved again in ethyl-acetate to obtain mass concentration 5 mg/mL. The scavenging activity of the extract was estimated by the percentage decrease of the relative intensity of the signal of DMPO-OH radical adduct with reference to the control without extract. The scavenger effect (SE) was calculated with the formula:

($\text {(1)) $$\begin{array}{c} \text{HRSE}=\left[\frac{h_{o}-h_{x}}{h_{o}}\right]\times 100\%, \end{array}$$

where *h*_0_ and *h*_*x*_ are heights of the second peak in ESR spectra of the control and sample, respectively.  Figure 1 presents ESR spectra of DMPO-OH in the presence of irradiated soybean extract.  Figure 2 presents the effect of different doses of gamma irradiation on HRSE of soybean extracts.

### 2.2. Quantitative Estimation of Isoflavones by HPLC

#### 2.2.1. Sample Preparation for Determination of Isoflavone Content

Two grams of dried, finely ground samples was placed in a 125-mL screw-top Erlenmeyer flask containing 10 mL of ACN and 2 mL of 0.1 N HC1 [3] and stirred for 2 h at room temperature. Extracts were filtered through Whatman no. 42 filter paper. The filtrate was taken to dryness on a rotatory evaporator at 45°C. The dried material was redissolved in 10 mL of 80% HPLC grade MeOH in water. An aliquot of sample was filtered through a 0.45-pm PTFE filter unit (poly(tetrafluoroethylene), Alltech Associates Inc., Deerfield, IL, USA) and analyzed by HPLC. 

#### 2.2.2. Conditions for HPLC

A linear HPLC gradient was composed of (A) 0.1% glacial acetic acid in H_2_O and (B) 0.1% glacial acetic acid in ACN. Following injection of 20 *μ*L of sample, solvent B was increased from 15% to 35% over 50 min and then held at 35% for 10 min. The solvent flow rate was 1 mL/min. A Waters 991 series photodiode array detector monitored from 200 to 350 nm. The minimum detectable concentrations for daidzein and genistein were 185 and 100 ng/mL, respectively. UVspectra were recorded and area responses were integrated by Waters PDA software.

### 2.3. Data Analysis

All procedures (extraction and measurements) were obtained from triplicate measurements. Statistical comparisons between samples were performed with Duncan *t*-test for independent observations. Differences were considered significant at *P* < 0.05. The antioxidant test results were investigated with multivariate analysis. The correlation matrix was calculated, giving the correlation coefficients between each pair of variables, that is, the analytical parameters tested. Each term of the matrix is a number ranging from −1 to +1; the + or − sign indicates a positive or negative interdependence between variables (direction), and the absolute value indicates the strength of the interdependence. Correlations between different parameters were considered significant at *r* > 0.95 (*P* < 0.05). Autoscaling transformation of data for antioxidant markers (genistin and daidzin contents, TIC, DPPH RSC, and HRSE) was done using STATISTICA.

## 3. Results and Discussion

### 3.1. Total Phenol, Tannin, and Isoflavone Contents

Data for total phenol and tannin contents in soybean were obtained from Štajner et al. [11]. All data including isoflavone contents were presented in Table 1. The highest increase of both phenolic parameters was found under dose at 1 kGy. Significant positive correlation between TPC and TTC was found (*r* = 0.8987).

Under the influence of gamma irradiation genistein content decreased from 21.19 (control) to 18.18 mg/kg (under dose of 10 kGy), daidzein content also decreased from 34.57 (control) to 29.00 mg/kg (10 kGy), genistin content increased from 368.2 (control) to 425.0 (10 kGy) mg/kg, and daidzin content increased from 242.1 (control) to 283.0 mg/kg (10 kGy). Total isoflavone content (TIC) gradually increased from 666.1 (control) to 755.2 mg/kg (10 kGy) (Table 1). Significant positive correlation was found between both isoflavone aglycones genistein and daidzein (*r* = 0.9664) and isoflavone glycosides (*r* = 0.8785). Total isoflavone content was positively correlated with both glycosides, genistin (*r* = 0.9831) and daidzin (*r* = 0.9496). High negative correlations were found between isoflavone glycosides and their aglycones.

The trend in total phenol content changes under the gamma irradiation in tested genotype Ana is consistent with the results obtained by Dixit et al. [10] where, after the initial increase (under 0.5 and 2 kGy), the decrease was observed under higher doses. We observed that in all irradiated samples TPC was higher than in nonirradiated control. TTC also increased due to gamma irradiation. There are numerous publications which confirm positive effect of ionizing radiation on the accumulation of phenolic compounds and therefore antioxidant activity in selected food and food materials [13–17]. Some studies also showed that low doses of gamma irradiation can increase in phenol compounds due to a spurt in the activities in key enzymes of phenylpropanoid metabolic pathway [18]. 

Dixit et al. [10] investigated three soybean genotypes with black, green, and yellow seed coat color and irradiated them with 0.5, 2 and 5 kGy with irradiation rate of 99.72 Gy/min. They observed the significant increase of genistein content at dose of 0.5 kGy and afterwards slightly decrease of the parameter and daidzein content was not significantly changed in two of three investigated genotypes. In our case, yellow coated seed genotype Ana responded to the irradiation up to 10 kGy (with dose rate of 228 Gy/min) by the decrease of free aglycones and the increase of glycosides. This profile of isoflavone constituents could be attributed to the conversion of malonyl-derivates into free glycosides or may be also the result from increased synthesis. Malonyl-derivates, which are always present in soybean seeds, are heat sensitive [19]. In our case, they were probably preserved because of the extraction which is carried out at room temperature and after gamma irradiation they were decomposed into demalonyl-glycosides. It can be assumed that changes of the content of isoflavones under irradiation depend on several factors such as genotype and irradiation conditions such as dose rate and extraction procedure of isoflavones.

### 3.2. DPPH RSC, FRAP, HRSE Determination

**Figure 3:**
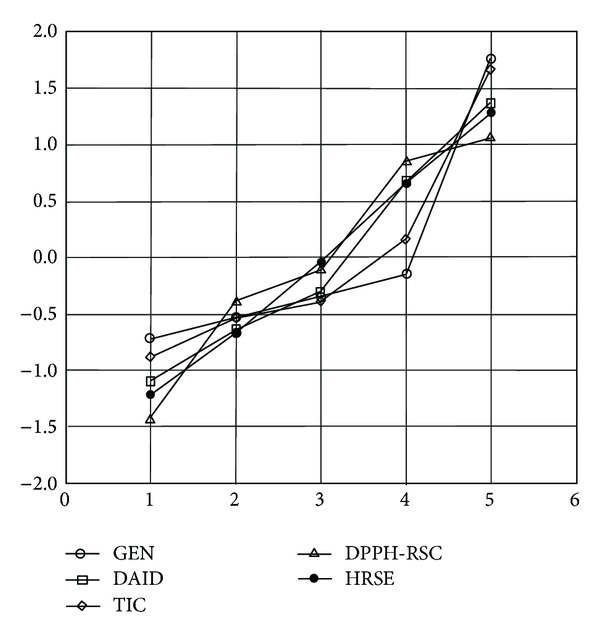
Autoscaling transformation of data for different antioxidant markers with significant positive correlations (genistin and daidzin contents, TIC, DPPH RSC, and HRSE); GEN (genistin); DAID (daidzin); TIC (total isoflavone contents); DPPH-RSC (DPPH radical scavenging capacity); HRSE (hydroxyl radical scavenging effect).

Data for DPPH RSC, FRAP, and HRSE are presented in Table 1. Significant positive correlations were found between DPPH RSC and HRSE. HRSE was also significantly positively correlated with TIC (*r* = 0.9182). Daidzin was significantly positively correlated with both parameters, DPPH RSC (*r* = 0.9581) and HRSE (*r* = 0.9873). Significant negative correlations were found between isoflavone aglycones (genistein and daidzein) and DPPH RSC and HRSE. Line plot of multiple variables after autoscaling transformation of positively correlated parameters (genistin, daidzin, TIC, DPPH-RSC, and HRSE) was presented by Figure 3.

Our result that Ferric reducing antioxidant power significantly increased at low level dose of 1 kGy was in accordance with results of Dixit et al. [10] and it can be explained that the FRAP parameter is mostly highly positively correlated with the total phenolic content [20]. However DPPH RSC and HRSE were highly positively correlated with genistin and daidzin contents and also with TIC. Although, some authors [21] hypothesized that the conversion of isoflavone glycosides into aglycones in soybean enhances the antioxidant capacity, other authors [22] showed that there was no correlation among aglycone forms of isoflavones in soybean and EPR signal of radiation induced free radicals. On the other hand, according to the investigation of Lee et al. [5], glycoside genistin had greater DPPH scavenging capacity than other isoflavones and glycosides including malonyl-derivates. According to Olivera et al. [22] there is also negative correlation among glycoside daidzin and EPR signal of radiationally formatted free radicals and appropriate antioxidant activity of daidzin. Bearing in mind before mentioned, it is easy to explain the increase of DPPH RSC and HRSE due to the increase of isoflavone glycosides. Total phenol and tannin contents are significantly positively correlated with FRAP, but just partly contribute to DPPH and ^•^OH radical scavenging capacities. The structural heterogeneity of flavonoids in different genotypes of soybean, with multiple mechanisms of action, the diverse methods used to evaluate their antioxidant activity, and different experimental conditions of irradiation may all lead to different responses of soybean under irradiation. 

## 4. Conclusion

Present results showed that gamma irradiation of soybean with doses up to 10 kGy enhanced HRSE and accumulation of genistin and daizin. Significant positive correlation between genistin, daidzin, DPPH-RSC, and HRSE was found and negative correlation between isoflavone glycosides and aglycones was found. Thus, besides antimicrobial activity, radiation induces antioxidant activity and therefore increases nutritional quality.

## Figures and Tables

**Table 1:** Effect of different doses of gamma irradiation on isoflavone (genistein, daidzein, genistin, and daidzin), total phenol and tannin contents, DPPH RSC, and FRAP of soybean extracts.

Dose (kGy)	Genistein (mg/kg)	Daidzein (mg/kg)	Genistin (mg/kg)	Daidzin (mg/kg)	TIC (mg/kg)	TPC (mg kat./g)**	TTC (mg kat./g)**	DPPH RSC (%)**	FRAP (FRAP units)**
0	21.19	34.57	368.2	242.1	666.1	2.204	0.968	40.26	1.35
1	21.47	34.51	372.4	249.8	678.2	2.423*	1.182*	42.74*	1.58*
2	19.77	31.73	376.5	255.1	683.1	2.319*	1.127*	43.40*	1.24
4	18.73*	31.38*	381.2*	271.4*	702.7	2.309*	1.103	45.71*	1.38
10	18.18*	29.00*	425.0*	283.0*	755.2*	2.382*	1.095*	46.20*	1.44

*Marked values are significantly different in comparison with nonirradiating control (Tukey test, *P* < 0.05).

**Data obtained from Štajner et al. [11].

TIC: total isoflavone content (sum of all 4 isoflavones); TPC: total phenol content; kat: katechine; TTC: total tannin content; DPPH RSC: DPPH radical scavenging capacity; FRAP: ferric reducing antioxidant capacity.
